# Resting Energy Expenditure of Master Athletes: Accuracy of Predictive Equations and Primary Determinants

**DOI:** 10.3389/fphys.2021.641455

**Published:** 2021-03-22

**Authors:** Petra Frings-Meuthen, Sara Henkel, Michael Boschmann, Philip D. Chilibeck, José Ramón Alvero Cruz, Fabian Hoffmann, Stefan Möstl, Uwe Mittag, Edwin Mulder, Natia Rittweger, Wolfram Sies, Hirofumi Tanaka, Jörn Rittweger

**Affiliations:** ^1^German Aerospace Center (DLR), Institute of Aerospace Medicine, Cologne, Germany; ^2^Experimental and Clinical Research Center – a joint co-operation between Charité Universitätsmedizin Berlin and Max Delbrück Center for Molecular Medicine, Berlin, Germany; ^3^College of Kinesiology, University of Saskatchewan, Saskatoon, SK, Canada; ^4^Facultad de Medicina, Instituto de Investigación Biomédica de Málaga, Universidad de Málaga, Málaga, Spain; ^5^Department of Internal Medicine III, University Hospital Cologne, Cologne, Germany; ^6^Department of Kinesiology and Health Education, The University of Texas at Austin, Austin, TX, United States; ^7^Department of Pediatrics and Adolsecent Medicine, Hospital Cologne, Cologne, Germany

**Keywords:** resting energy expenditure, master athletes, energy metabolism, predictive equation, body composition

## Abstract

Resting energy expenditure (REE) is determined mainly by fat-free mass (FFM). FFM depends also on daily physical activity. REE normally decreases with increased age due to decreases in FFM and physical activity. Measuring REE is essential for estimating total energy expenditure. As such, there are a number of different equations in use to predict REE. In recent years, an increasing number of older adults continue to participate in competitive sports creating the surge of master athletes. It is currently unclear if these equations developed primarily for the general population are also valid for highly active, older master athletes. Therefore, we tested the validity of six commonly-used equations for predicting REE in master athletes. In conjunction with the World Masters Athletic Championship in Malaga, Spain, we measured REE in 113 master athletes by indirect calorimetry. The most commonly used equations to predict REE [Harris & Benedict (H&B), World Health Organization (WHO), Müller (MÜL), Müller-FFM (MÜL-FFM), Cunningham (CUN), and De Lorenzo (LOR)] were tested for their accuracies. The influences of age, sex, height, body weight, FFM, training hours per week, phase angle, ambient temperature, and athletic specialization on REE were determined. All estimated REEs for the general population differed significantly from the measured ones (H&B, WHO, MÜL, MÜL-FFM, CUN, all *p* < 0.005). The equation put forward by De Lorenzo provided the most accurate prediction of REE for master athletes, closely followed by FFM-based Cunningham’s equation. The accuracy of the remaining commonly-used prediction equations to estimate REE in master athletes are less accurate. Body weight (*p* < 0.001), FFM (*p* < 0.001), FM (*p* = 0.007), sex (*p* = 0.045) and interestingly temperature (*p* = 0.004) are the significant predictors of REE. We conclude that REE in master athletes is primarily determined by body composition and ambient temperature. Our study provides a first estimate of energy requirements for master athletes in order to cover adequately athletes’ energy and nutrient requirements to maintain their health status and physical performance.

## Introduction

**FIGURE 1:**
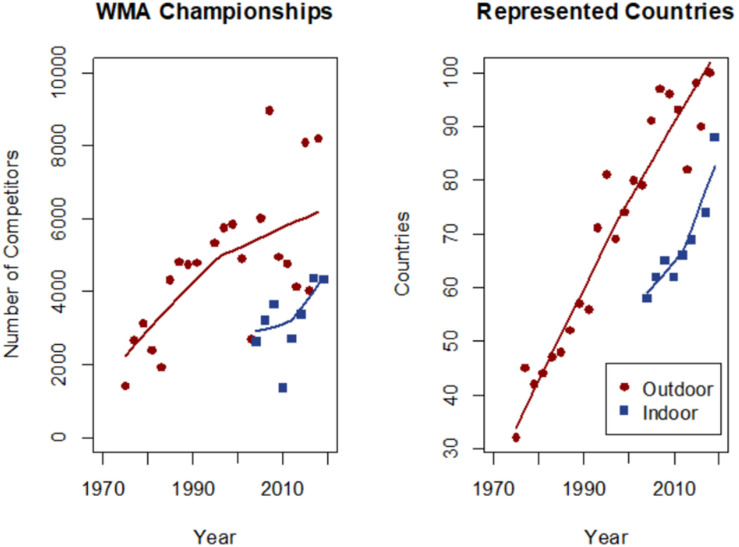
Participation in world masters athletics championship in the past 4 decades, quantified by the number of competing athletes (left) and by the number of countries represented (right). Data are courtesy by the World Master Athletics (WMA).

Master athletes are individuals older than 35 years who continue physical training and take part in athletic competitions, often throughout their entire life, which can be regarded as a model of active aging (Tanaka et al., 2019). Over the past decades, the number of master athletes has increased steadily (Figure 1). Because of their strenuous training routine and specific demands that age-related physiological changes place, master athletes have distinct nutritional requirements (Desbrow et al., 2019). A sufficient energy intake is mandatory to maintain overall health and competitive performance of these aging athletes (Schofield et al., 2019). Resting energy expenditure (REE), the fraction of energy at rest to maintain essential body functions, contributes 60–70% to total daily energy expenditure (TEE). REE is determined by fat-free mass (FFM) (Sullo et al., 2004; Schofield et al., 2019). FFM accounts for 60–70% of the total REE, and fat mass (FM) for only 5–7% of REE (Johnstone et al., 2005). REE declines with increasing age, which is thought to be primarily driven by the gradual decline in FFM due to a loss of muscle mass (Tzankoff and Norris, 1977; Welle and Nair, 1990). Additionally, albeit to a lesser extent, reduced physical activity contributes to this decline in REE (Poehlman et al., 1991a,b,c; Vaughan et al., 1991). Master athletes can theoretically counteract such age-related decreases in FFM and thereby in REE, provided that energy intake levels are sufficient. Having valid and reliable REE values is crucial to establish achievable goals for dietary and exercise interventions involving master athletes (Amaro-Gahete et al., 2019). Indirect calorimetry (IC) has been established as a non-invasive method for measuring REE with good precision and accuracy (Haugen et al., 2007; Pinheiro Volp et al., 2011; Delsoglio et al., 2019). IC is based on measuring oxygen (O_2_) consumption and carbon dioxide (CO2) dissipation, which is coupled to the metabolism of energy-rich substrates such as carbohydrates and fats. Total average daily REE in kcal was calculated using the modified Weir equation (Weir, 1949) as follows:

$$\text{REE}\left(\mathrm{kcal}/\mathrm{day}\right)=[\left({\mathrm{VO}}_{2}\times 3.941\right)+\left({\mathrm{VCO}}_{2}\times 1.11\right)+\left({\mathrm{uN}}_{2}\times 2.17\right)]\times 1,440.$$

The urinary nitrogen component (uN_2_) is often excluded when calculating EE because it only accounts for around 4% of the true energy expenditure. It contributes only to a small error of 1–2% in the calculation of final REE in both inpatients and outpatients. Thus, the abbreviated equation below is commonly used (Ferrannini, 1988).

$$\mathrm{REE}\,\left(\mathrm{kcal}/\mathrm{day}\right)=\left[\left({\mathrm{VO}}_{2}\times 3.941\right)+\left({\mathrm{VCO}}_{2}\times 1.11\right)\right]\times 1,440.$$

However, the IC technique is time-consuming, expensive, and, unavailable in daily practice. Accordingly, REE as well as TEE are often predicted via one of various prediction equations (Vander Weg et al., 2004; ten Haaf and Weijs, 2014; Cherian et al., 2018; Amaro-Gahete et al., 2019; Schofield et al., 2019). The Harris & Benedict (H&B), World Health Organization (WHO), Müller (MÜL), Müller-FFM (MÜL-FFM) and Cunningham (CUN) equations are most commonly used, but none of these standard prediction equations fits for every individual. While H&B, WHO, MÜL only take age, sex, body weight and/or height into account, the equations by Müller-FFM and Cunningham also use FFM as a contributing factor (Harris and Benedict, 1918; Cunningham, 1980; WHO, 1985; De Lorenzo et al., 1999; Müller et al., 2004). Given that all predictive equations include age as covariate and that the underlying study cohorts likely only involved relatively sedentary older adults, it remains unclear whether these predictive equations would be accurate for highly active, older master athletes. To further highlight this concern, the accuracy of REE equations has already been questioned for athletic populations in general (Thompson and Manore, 1996; De Lorenzo et al., 1999; ten Haaf and Weijs, 2014; Jagim et al., 2018; Schofield et al., 2019) because data concerning subjects’ physical activity involved in the development of the various equations were not considered. Studies on young athletes show increased energy requirements, but also emphasize substantial individual differences depending on the type of exercise activity (Frączek et al., 2019). The most widely-used equation for athletes comes from De Lorenzo (De Lorenzo et al., 1999). These authors studied seven published REE equations for estimating REE in young male athletes, aiming to create a specific equation for male athletes. Currently, the American College of Sports Medicine (ACSM) recommends the prediction equations developed by Cunningham and, interestingly, also the one by Harris & Benedict for estimating REE in athletes (Rodriguez et al., 2009), although these equations have not been developed for the athletic population. More importantly, the accuracy of REE prediction models has not been validated specifically for these populations, and certainly not for master athletes. The number of studies measuring REE in older athletes is small. Available studies suggest that older men who are competitively physically active have a higher REE compared with age-matched sedentary individuals (Poehlman and Horton, 1990; Campbell et al., 1994). This is confirmed by a later study (Ryan et al., 1996; Van Pelt et al., 1997) that REE was greater in both young and older female athletes than in sedentary controls of the same age. A significant increase in REE was also observed in older female athletes after a short training phase of only 6 weeks (Stavres et al., 2019).

Thompson & Manore (Thompson and Manore, 1996) reported that traditionally-used equations for estimating metabolic rates do not apply well to about 50% of athletes and that the Cunningham equation provides an accurate estimate of REE when determining energy needs of highly active people. Equations taking FFM into account seem to be the most reasonable approach to predict REE in athletes (ten Haaf and Weijs, 2014). However, De Lorenzo failed to determine FFM as the best predictor of REE for male athletes, instead the combination of height and body mass were the best predictors (De Lorenzo et al., 1999).

With this information as background, the purpose of this study was to test the accuracy of six common equations for predicting REE in master athletes and to address which other anthropometric and environmental characteristics influence REE in this specific cohort. We hypothesized that most of the existing equations would underestimate REE because of the higher FFM proportion in master athletes vs. sedentary adults.

## Materials and Methods

### Study Setting and Subjects

The Masters Athletic Field Study 2018 (MAFS-18) aimed to assess physiological and mental constituents of physical fitness and well-being in master athletes. The present investigation is a sub-study of the MAFS-18, which was implemented during the 23rd World Masters Athletics Championship in September 2018 in Malagà, Spain. Any who was admitted to the championship as a competing athlete was allowed to participate. Exclusion criteria were injuries or illnesses that affected jump tests or contraction of the calf muscle, another main outcome measure of the MAFS-18. Criteria for early termination of the study were pathological findings during measurements or voluntary termination by participants. Athletes were made aware of the study in advance by direct e-mails and posters. The study protocol was approved by the ethical committee of the North Rhine Medical Association (Ärztekammer Nordrhein lfd Nr. 2018171), and the study had been registered on the German register for clinical trials^1^ with registration number DRKS00015172. All the participants signed informed consent to participate in the present study.

A hundred and thirteen athletes (79 men, 34 women), 35–84 years old, were studied. Athletes were asked to fast overnight, at least 12 h, before the test session. They were requested to minimize high intensity physical activity or competitions 24 h before testing.

### Experimental Design

The MAFS-18 study is a field study in an analytical cross-sectional design, performed once under field conditions. The study flow involved to first sign in at the registration office, the collection of anthropometric and training data, and body composition. Then, an appointment was made for a subsequent day to take REE-measurements.

### Measurements

All the measurements were conducted in the morning between 7.00 and 11.00 am in a dedicated room of the main stadium close to the registration office (room temperature 27.6 ± 1.9°C, ambient pressure 765 mmHg, ambient humidity 60%).

### Anthropometric Measurements

A stadiometer was used for assessing height to the nearest of 0.1 cm, body weight was measured by a calibrated balance scale with an accuracy of 100 g, both measured standing.

### Body Composition

Body composition was measured by a segmental multi-frequency bioelectrical impedance analyzer (BIA) (InBody S10, Eschborn, Germany), following manufacturer’s instructions. The electrodes were attached to the participant in supine position on the right hand and right foot with legs apart. Resistance and reactance were determined using an electric alternating current of 800 mA and multiple frequencies of 5, 50, and 250 kHz. Body composition indicators including skeletal muscle mass (SMM), soft lean mass (SLM), percentage of body fat (PBF), fat free mass (FFM), fat mass (FM), intracellular water (ICW), extracellular water (ECW), total body water (TBW), mineral content and protein content were measured. Phase angle at 50 kHz was calculated by using the equation: phase angle (°) = arctan (reactance/resistance) x (180/π).

### Indirect Calorimetry

Resting energy expenditure was measured by indirect calorimetry using a canopy device (Quark RMR, COSMED Deutschland GmbH, Fridolfing, Germany), following manufacturer’s instructions and settings. After gas- and flowmeter-calibration, a two-minute REE test-measurement was conducted, before the main measurement over 30 min started. Oxygen consumption (VO_2,_ ml/min) and carbon dioxide production (VCO_2_, ml/min) were measured for calculating REE. Indirect calorimetry was performed in the morning between 7.00 and 11.00 a.m. after an overnight fast in a separate corner of the examination room with an ambient temperature of 27.6 ± 1.9°C. The minimum duration of measurement was 30 min and the first 5 min were discarded. During measurements, subjects had to remain motionless, were not allowed to speak, or fall asleep. A protocol was taken whether they moved or felt asleep. Corresponding data points were excluded from the analysis. The device collected a measured value every 10 s for 30. The flow rate under the hood was adjusted so that the expiratory air (FeCO2) was between 0.9–1.0 l and the urinary nitrogen value was set to 8.00064 g/day. Data were collected every 10 s for 30 min. The experimental conditions were standardized for each subject by means of a checklist. The CVs for the repeated REE-measurements were 13% in and 14% in women.

### Data Processing

The first 5 min and some outliers of the indirect calorimetry data collection were removed and average values of VO_2_ and VCO_2_ were taken to calculate REE and respiratory exchange ratio (RER) as previously described (Ferrannini, 1988).

Directly-measured REE values were compared with a variety of REE values predicted based on anthropometric data. Equations of Harris & Benedict (H&B) (Harris and Benedict, 1918), WHO (WHO, 1985), Müller et al. (MÜL) (Müller et al., 2004), Müller-FFM (MÜL-FFM) (Müller et al., 2004), Cunningham (CUN) (Cunningham, 1980), and De Lorenzo et al. (LOR) (De Lorenzo et al., 1999) were used for comparison.

Harris & Benedict defined REE as an individual’s heat production assessed 12–14 h after the last meal at rest (Harris and Benedict, 1918). The underlying cohort consisted of 333 subjects, and height, age, body weight, and sex were reported as strongest predictors of REE:

M⁢e⁢n:R⁢E⁢E⁢(k⁢c⁢a⁢l/d)=66.47+13.75×B⁢o⁢d⁢y⁢w⁢e⁢i⁢g⁢h⁢t⁢(k⁢g)+5.0×H⁢e⁢i⁢g⁢h⁢t⁢(c⁢m)-6.76×A⁢g⁢e⁢(y⁢e⁢a⁢r⁢s).

W⁢o⁢m⁢e⁢n:R⁢E⁢E⁢(k⁢c⁢a⁢l/d)=655.1+9.56×B⁢o⁢d⁢y⁢w⁢e⁢i⁢g⁢h⁢t⁢(k⁢g)+1.85×H⁢e⁢i⁢g⁢h⁢t⁢(c⁢m)-4.68×A⁢g⁢e⁢(y⁢e⁢a⁢r⁢s).

World Health Organization equation were calculated using several studies aimed at estimating the food requirements of certain populations by measuring REE and physical activity (WHO, 1985). A total of 7,549 subjects were measured and additional mean REEs of 3,874 other study measurements were included to define prediction equations including sex, body weight and age as greatest influencing factors:

M⁢e⁢n⁢ 30-60⁢y⁢e⁢a⁢r⁢s:R⁢E⁢E⁢(k⁢c⁢a⁢l/d)=11.6×B⁢o⁢d⁢y⁢w⁢e⁢i⁢g⁢h⁢t⁢(k⁢g)+879.

M⁢e⁢n>60⁢y⁢e⁢a⁢r⁢s:R⁢E⁢E⁢(k⁢c⁢a⁢l/d)=13.5×B⁢o⁢d⁢y⁢w⁢e⁢i⁢g⁢h⁢t⁢(k⁢g)+487.

W⁢o⁢m⁢e⁢n⁢ 30-60⁢y⁢e⁢a⁢r⁢s:R⁢E⁢E⁢(k⁢c⁢a⁢l/d)=8.7×B⁢o⁢d⁢y⁢w⁢e⁢i⁢g⁢h⁢t⁢(k⁢g)+829.

W⁢o⁢m⁢e⁢n>60⁢y⁢e⁢a⁢r⁢s:R⁢E⁢E⁢(k⁢c⁢a⁢l/d)=10.5×B⁢o⁢d⁢y⁢w⁢e⁢i⁢g⁢h⁢t⁢(k⁢g)+596.

Müller et al. (2004) compared equations of WHO with new data of a compiled German database, where REE measurements were conducted on 2,528 German participants to specify the WHO equations for the German population. He defined two different equations, one containing body composition data including fat free mass (FFM) and fat mass (FM):

REE(kcal/d)=[0.047×Bodyweight(kg)+1.009

×sex(0=female,1=male)-0.01452×age(years)+3.31]×1,000/4.184.

REE(kcal/d)=[0.05192×FFM(kg)+0.04036×FM(kg)

+0.869×sex(0=female,1=male)-0.01181×age(years)+2.992]×1,000/4.184.

Cunningham solidified Harris & Benedict’s idea that FFM could be the greatest influencing factor on REE and developed an equation with FFM as the only parameter with participants from the Harris & Benedict’s study (Cunningham, 1980):

R⁢E⁢E⁢(k⁢c⁢a⁢l/d)=500+22×F⁢F⁢M⁢(k⁢g).

De Lorenzo et al. (1999) defined an equation specifically for athletes. REE of 51 young (age 23.3 ± 3.5 years) male subjects, who exercised regularly at least 3 h per day was measured and the following equation was derived:

R⁢E⁢E=-857+9×B⁢o⁢d⁢y⁢w⁢e⁢i⁢g⁢h⁢t⁢(k⁢g)+11×H⁢e⁢i⁢g⁢h⁢t⁢(c⁢m).

### Statistical Analyses

Data were collected with Research Electronic Data Capture with (REDCap^®^). Statistical Package for Social Sciences (SPSS Version 26, IBM) was used for all statistical analyses. At first, all parameters were tested for normality by Shapiro-Wilk-Test. Non-parametric Mann-Whitney-U-Test was selected for comparison of estimated and measured REE. Bland-Altman analysis was selected to assess differences between measured and predicted REE. The differences between the measured and predicted values were set against their mean values, and the limits of agreement were defined. A twofold standard deviation is accepted as the tolerance limit, as it should cover 95% of all data. Linear regression analysis was used to evaluate the REE-FFM association. Multiple-regression analysis was used to evaluate independent relationships between REE and sex, age, height, body weight, FFM, training hours per week, phase angle, and athletic specialization. Sex and sport speciality (endurance, power, or mixed) represent categorical variables, which first needed to be encoded. Female sex and power as sports were chosen as reference categories, which allows a quantitative comparison of all categories with the reference categories. Significance level for all tests was set at 0.05. Subjects characteristics and environmental data are expressed as mean ± SD, all other results as mean ± SEM.

## Results

Subject characteristics’ are presented in Table 1.

**TABLE 1:** Subjects characteristics’.

	Men	Women
Age (years)	57.14 ± 11.65 (35–84)	54.91 ± 11.60 (35–80)
Height (cm)	174.86 ± 7.25 (160.0–197.1)	164.95 ± 5.99 (153.1–178.8)
Weight (kg)	74.24 ± 10.31 (56.0–100.7)	62.12 ± 9.97 (45.7–91.4)
BMI (kg/m^2^)	24.22 ± 2.46 (18.93–32.29)	22.79 ± 3.26 (18.54–32.17)
FFM (kg)	60.75 ± 8.36 (48.0–88.9)	48.20 ± 6.41 (36.6–63.5)
FFM (%)	81.84 ± 6.10 (71.3–94.1)	77.72 ± 6.75 (54.9–91.5)
FM (kg)	13.71 ± 5.50 (3.5–32.6)	14.27 ± 6.50 (5.9–38.8)
FM (%)	18.16 ± 6.10 (7.8–32.4)	22.28 ± 6.75 (8.5–54.1)
Training (h/w)	8.50 ± 4.60 (1.0–30.0)	9.91.50 ± 6.66 (1.0–30.0)

### Comparison of Measured REE and Predicted REE

**FIGURE 2:**
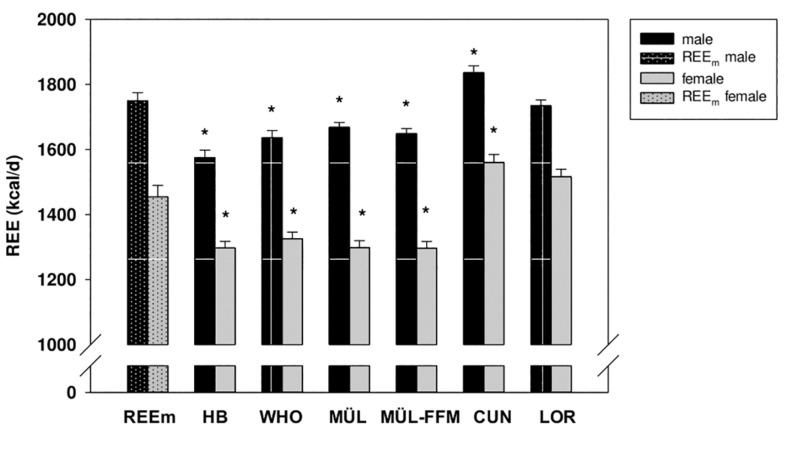
Directly-measured resting energy expenditure (REE) (REEm) and predicted REE (REEp) using a variety of equations including Harris & Benedict (H&B), World Health Organization (WHO), Müller (MÜL), Müller-FFM (MÜL-FFM), Cunningham (CUN), and De Lorenzo (LOR) in males and female master athletes. *p < 0.005 vs. REEm.

As illustrated in Figure 2, predicted REEs (REEp) using equations created for the general population differed significantly from the directly-measured REE (REE_m_) (*p* < 0.001 for H&B; *p* = 0.001 for WHO; *p* = 0.004 for MÜL; *p* = 0.004 for MÜL-FFM; *p* = 0.003 for CUN). More specifically, the equations H&B, WHO, MÜL, and MÜL-FFM underestimated, and CUN overestimated REE in both sexes. Predicted REE by LOR was not significantly different from the measured REE (*p* = 0.750).

For male athletes, mean differences between measured and predicted REE were smallest for LOR with 15.3 ± 17.7 kcal/d (1% deviation from the measured REE) followed by CUN with −76.8 ± 18.4 kcal/d (−4% deviation), MÜL with 81.7 ± 18.0 kcal/d (5% deviation), MÜL-FFM with 110.6 ± 17.7 kcal/d (7% deviation) and WHO with 113.6 ± 19.9 kcal/d (8% deviation). The mean difference is highest for H&B with 174.56 ± 18.56 kcal/d (12% deviation).

For women, mean differences between measured and predicted RER were also smallest for LOR with −62.0 ± 26.7 kcal/d (−4% deviation from the measured REE) followed by CUN with −99.8 ± 23.2 kcal/d (−6% deviation) and WHO with 128.9 ± 28.1 kcal/d (10% deviation). The mean difference is highest for MÜL with 156.1 ± 7.0 kcal/d (12% deviation), H&B with 157.5 ± 26.5 kcal/d (12% deviation) and MÜL-FFM with 164.5 ± 26.8 kcal/d (13% deviation).

**FIGURE 3:**
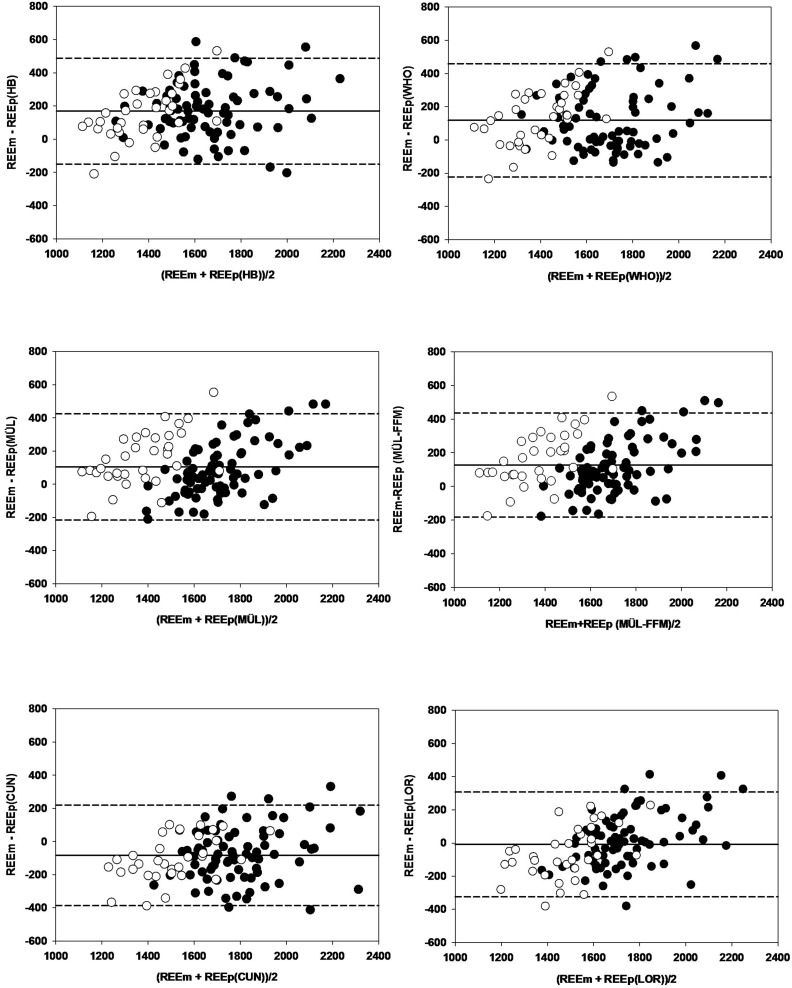
Bland-Altman plots for six [Harris & Benedict (HB), WHO (WHO), Müller (MÜL), MÜL -FFM (MÜL-FFM) Cunningham (CUN), De Lorenzo (LOR)] resting energy expenditure (REE) predictive equations (REEp). The solid lines represent the mean difference (BIAS) between predicted and measured REE (REEm). The upper and lower dashed lines represent 95% limits of agreement. • female ∘ male.

Figure 3 shows Bland-Altman plots for the six selected equations in men and women. The limits of agreement, based on the Bland-Altman plots, show the interval of two standard deviations of the measurement differences either side of the mean difference and were large in all six cases. For men, the De Lorenzo equation showed the smallest range with 615.2 kcal/d. For women, the Cunningham equation showed the smallest range with 523.3 kcal/d (Table 2).

**TABLE 2:** Limits of agreement.

	Limits of agreement (range) (kcal/d)	
	
	Men	Women
Harris & Benedict	−148.84 to 497.96 (646.79)	−144.81 to 459.72 (604.54)
WHO	−233.16 to 460.37 (693.53)	−192.06 to 449.86 (641.86)
Müller	−231.43 to 394.86 (626.29)	−151.96 to 464.20 (616.16)
Müller-FFM	−139.91 to 415.16 (609.07)	−137.44 to 466.50 (603.93)
Cunningham	−393.04 to 239.50 (632.54)	−361.05 to 162.25 (523.30)
De Lorenzo	−292.28 to 322.93 (615.21)	−366.87 to 242.84 (609.71)

Table 3 shows the percentage of accuracy and the percentage of under- and overestimation of REE predictive equations. The De Lorenzo equation resulted in the highest percentage accurate predicted equations for men (72.2%), the Cunningham equation for women (63.6%). The Harris & Benedict equation showed in both sexes less than 50% accuracy. The WHO and the MÜL equations showed less than 50% accuracy only in women.

**TABLE 3:** Accuracy of predictive equations.

	Underestimation (%)		Accurate estimation (±10%)		Overestimation (%)	
			
	Men	Women	Men	Women	Men	Women
Harris & Benedict	51.9	50.0	48.1	47.1	0	2.9
WHO	36.7	52.9	63.3	41.2	0	5.9
Müller	27.9	50.0	65.8	47.1	6.3	2.9
Müller-FFM	32.5	51.5	66.2	45.5	1.3	3.0
Cunningham	6.6	0	68.4	**63.6**	25.0	36.4
De Lorenzo	17.7	11.8	**72.2**	61.8	10.1	26.5

*Bolded values denotes that, this is the most accurate estimation in men and in women.*

### Linear Regression Analysis for REEm/FFM Ratio

**FIGURE 4:**
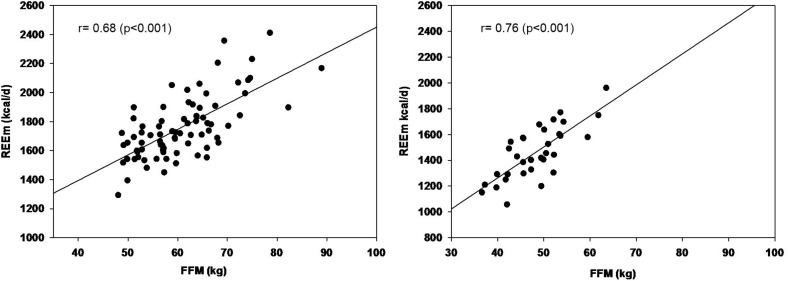
Relationship between the measured resting energy expenditure (REEm) and fat free mass (FFM) of master athletes. On the left: men (*r* = 0.69, *p* < 0.001), on the right: women (*r* = 0.76, *p* < 0.001).

The relationship between REE and FFM was evaluated by linear regression. In the simple linear model, the FFM has a significant influence on the REE (*p* < 0.001) (Figure 4). The contribution of FFM to REE was calculated as 29.17 ± 0.34 kcal/kg FFM for men and 30.42 ± 0.52 for women.

### Multiple Regression Analyses

For multiple regression analyses, six participants were excluded due to missing body composition measurements (2 men, 1 woman), no indication of the sporting event (1 man), or no indication of the training hours per week (1 man, 1 woman), so the remaining 107 (75 men, 32 women) participants were included for the multiple regression analysis.

Body weight, age, sex, height, FFM, FM, training hours per week, phase angle, ambient temperature, and athletic specialization, assessed via their self-rated best event, (endurance: *n* = 31, Power: *n* = 57, mixed: *n* = 19) were selected as predictor variables. The results of different models are given in Table 4. Body weight as the only predictor can explain 55.7% of the variance (*p* < 0.001, Model 1), while FFM as the only predictor can explain 63.0% of the variance (*p* < 0.001, Model 2). All other predictors increase the explained variance only slightly. A hierarchical analysis of all measured predictors selects the best combination of variables, which is shown in Model 7. FFM (*p* < 0.001), FM (*p* = 0.007), temperature (*p* = 0.004) and gender (*p* = 0.045) are the only significant predictors of REE in this model, they explain 69.0% of the variance. By extending the model by anthropometric and sport-specific predictors, the explained variance increases only slightly. These variables have only small influence on REE and are therefore only partially suitable for predicting it.

**TABLE 4:** Multiple regression model to determine independent predictors of resting energy expenditure (REE) (kcal/d).

Equation number	Tested variables	Equation	Explained variance (R^2^)
Model 1	Body weight	REE = 505.233 + 16.372 × body weight (kg)	55.7%
Model 2	FFM	REE = 494.873 + 20.623 × FFM (kg)	63.1%
Model 3	FFM, FM	REE = 439.574 + 20.462 × FFM (kg) + 4.645 × FM (kg)	64.2%
Model 4	FFM, FM, gender, age, height	REE = 431.859 + 17.6 × FFM (kg) + 5.462 × FM (kg) + 73.951 × sex (female = 0, male = 1) – 0.602 × age (years) + 0.821 × height (cm)	65.3%
Model 5	FFM, FM, gender, age, height, temperature	REE = −99.565 + 19.664 × FFM (kg) + 5.761 × FM (kg) + 62.716 × sex (female = 0, male = 1) −0.308 × age (years) – 0.610 × height (cm) + 23.531 × temperature (°C)	68.0%
Model 6	FFM, FM, gender, age, height, temperature, training hours, phase angle, kind of sports*	REE = −131.956 + 18.823 × FFM (kg) + 6.994 × FM (kg) + 77.484 × sex (female = 0, male = 1) + 0 × age (years) −0.567 × height (cm) + 22.550 × temperature (°C) + 0.007 × training hours (hours) + 7.104 × phase angle (°) + 24.245 × endurance – 9.736 × mixed *based on strength as reference	69.3%
Model 7	FFM, FM, temperature, gender	REE = −202.088 + 18.577 × FFM (kg) + 6.753 × FM (kg) + 23.910 × temperature (°C) + 78.479 × sex (female = 0, male = 1)	69.0%

One of the next steps could be to simplify the model by excluding all non-significant independent variables, in order to obtain a new predictive equation especially for master athletes to be tested and validated in independent cohort of master athletes.

REE = −202.088 + 18.577 × FFM (kg) + 6.753 × FM (kg) + 23.910 × temperature (°C) + 78.479 × sex (0 = female, 1 = male).

## Discussion

The main aims of this study were to assess the accuracy of existing predictive equations for REE applied to master athletes and to ascertain the primary determinants of REE in this group of athletes. The data presented herein show underestimation errors in most predicting equations by Harris & Benedict (12%), WHO/FAO (8%) and Müller (5 and 7% without and with FFM, respectively), whereas Cunningham’s equation overestimated the actual REE by 4%. Whilst predictions errors in the order of magnitude of 5% may seem acceptable in many other areas of research, they are of concern when it comes to providing dietary recommendations based on energy expenditure. Very clearly, overestimating the required caloric intake by such amounts will lead to obesity in the long run, and underestimation to starvation. The only equation that provided an accurate prediction on average was the one by De Lorenzo et al. (De Lorenzo et al., 1999), which is explained by the fact that it was specifically made for athletes. However, even though De Lorenzo’s REE prediction may have been acceptable on average, it still over- or underestimated in 30% of master athletes.

The Harris & Benedict equation, which is probably the most widely used approach in the clinical settings, resulted in about 50% of cases in accurate estimation of REE, in about 50% of cases in underestimation, but almost no overestimation in both men and women. The WHO and Müller equations (using body weight and FFM) resulted in about two thirds of men and almost 50% of women with accurate estimation of REE, about one third on men and about 50% on women with underestimation, but only in a few percent in overestimation. The Cunningham equation also resulted in about two thirds of both men and women with accurate estimation of REE, in less than 20% of cases in underestimation, but in more than 20% of cases a clear overestimation, specifically in women.

In general, our results of higher REE in master athletes are in accordance with the findings in other cross-sectional studies performed in young athletes (Ballor and Poehlman, 1992; Poehlman et al., 1992). The multiple regression analysis shows that this is mainly due to higher FFM in master athletes, compared with that of the general population. A previous study (ten Haaf and Weijs, 2014) pointed out that the Cunningham and De Lorenzo equations predicted REE in athletes more accurate than other commonly-used REE predictive equations (e.g., Harris & Benedict, WHO, Schofield, Mifflin, Owen), with the De Lorenzo equation being slightly less accurate than the Cunningham equation. It should, however, be noted that these notions were based on data of recreational athletes of 18–35 years (ten Haaf and Weijs, 2014) and were not based on data obtained from highly trained master athletes as presented in this study.

Interestingly, the De Lorenzo’s equation is the only predictive equation that was constructed based on a population of athletes. In their study, seven published REE equations were compared to assess the validity and reliability of REE estimations, based on a population of 51 young male athletes (22 water polo, 12 judo, and 17 karate) with a secondary aim of creating a specific equation for male athletes. In an attempt to improve the accuracy of the predictive equation, De Lorenzo et al. (De Lorenzo et al., 1999) incorporated body composition constituents, such as lean and fat mass obtained from DEXA scans. Contrary to expectations, FFM was not found to be the best predictor of REE, but rather a combination of height and body mass.

It is also of interest that the present study only found body weight, FFM, FM and sex as significant anthropometric predictors, but not age and height. This is noteworthy for two reasons. First, the absence of significant age effect suggests that master athletes maintain relatively high levels of basal metabolic rate, which also encompasses energy consumption as per repair and remodeling processes. Second, the finding of a significant contribution by FFM, FM and indicates that age effects by the previous predicting equation could be explained by age-related increases in body fat percentage.

Finally, we also checked the effect of ambient temperature. The average value during testing within the present study was 27.5°C which is higher than the above the temperature of 20–25°C that is recommended for the IC measurement by the manufacturer. Naturally, RMR assessments should normally be performed at a standard temperature, or at least controlled for it. Moreover, 5°C spread in this range seems *per se* quite wide, given the significant temperature effects observed in the present study. Unfortunately, the published literature neglects the temperature effect, and there is, to the best of our knowledge, only one study (Abreu-Vieira et al., 2015) that has investigated the effects of ambient temperature as influence factor on REE in mice.

Looking at the consequences that the elevated ambient temperature of the Málaga measurements may have had for the main study outcome, it is unfortunate that previous researchers had failed to analyze the effect of the ambient temperature information during their REE assessments. However, some simple calculations with the regression equation obtained from the present data suggest an excess REE by 23.91 kcal/day can be attributed to each 1°C-increase in ambient temperature (Table 4), and that therefore a deviation by 5 × 23.91 ∼120 kcal could be cause by elevating ambient from 22.5°C to 27.5°C. This amount is certainly a sizeable confounder, and it could potentially explain a good fraction of the excess REE observed in the present cohort, if not all of it. However, one also needs to consider that such model calculations involve extrapolation to ambient temperature levels below the range for which we have data, and that no information exists about temperature effects on REE in the 20–25°C range. Therefore, and given that the DeLorenzo equation predicts higher REE in athletes than non-athletes, we still hold it likely that athletic participation is also associated with increased REE levels at old age.

The present study shows that the De Lorenzo equation, by taking height and body weight into account, is remarkably similar to the Cunningham equation that takes the athlete’s FFM into account. These results are in accordance with the result of our multiple regression analysis that body weight as well as FFM are both significant predictors of REE in master athletes. One explanation for this equality in predictiveness could be the lower fat mass of the master athletes (18.2 ± 6.1% in men and 22.3 ± 6.8% in women) in comparison to the general elderly population (Deurenberg et al., 1989; Ofenheimer et al., 2020). A lower fat mass would effectively mean that FFM makes up a larger proportion of the total body mass in master athletes than in comparable non-exercising populations. This is supported by the comparison of the two Müller equations, which show similar results despite different predicators. While MÜL only uses the total body mass, MÜL-FFM additionally includes FFM. Though important determinants, body weight and FFM are not the sole predictors of REE; despite the inclusion of the FFM, for instance, the Müller equation showed less accuracy in predicting REE compared with the Cunningham equation. Since Müller et al. (2004) developed their equation primarily for the German, predominantly Caucasian population, the differences are potentially due to ethnic differences, which would require further investigation. However, it is interesting that the Bland-Altman Plots of the Müller equation show two separate clusters for men and women, which were not observed within the other equations.

A bias ranging from −587 kcal/d to 0 kcal/d was observed when the REE was predicted in master athletes. The question of what is an acceptable bias, needs to be evaluated from a practical perspective and depends on the individual training situation. However, the primary goal should be to avoid an energy deficiency in master athletes relative to what can be achieved through individual dietary advice.

The championship setting offered a unique opportunity to measure REE in many competitive master athletes over a short period with a modern, non-invasive technique. This was met by high interest and demand by master athletes participating in the Championship (*n* = 113). Unfortunately, this strength was also a limitation regarding the environmental conditions. There was only one test room for all measurements available in the stadium, although an REE-measurement requires a calm atmosphere. Ambient temperature (25–31°C) and humidity were relatively high (43–70%), which made calm breathing under the canopy difficult for some of the athletes. Unfortunately, day-to-day variations of temperature and humidity were unavoidable and reduced the required standardization. Independent of the environmental conditions, the age range (34–84 years) was large within the participants and the menopausal status was not considered in the evaluation (Van Pelt et al., 1997). In terms of the multiple linear regression, some variables affected others (e.g., FFM affecting body weight), which can lead to multicollinearity. On the other hand, this is hard to avoid when analyzing anthropometric data. Despite all being master athletes, the participants were heterogeneous in terms of the different sports disciplines and the associated FFM. For further investigations, it may be useful to stratify more by age, height, sex and sports to define the influence of FFM on REE. In this context, it would also be desirable to measure the FFM with devices that are specifically validated for athletes. In order to normalize the REE data for organ and tissue masses according to Bosy-Westphal et al. (2013) and Muller et al. (2018) MRI data of master athletes would be of great interest for future studies.

In summary, it is important to be careful when using REE equations in athletic individuals, given the body compositional differences between athletic and non-exercising populations. Typically, REE will be underestimated with REE equations that do not account for FFM and are not specifically developed for an athletic population. For the special collection of older competitive athletes, the present study provides an initial overview of their energy requirements. Further investigations under more standardized study conditions (e.g., controlled environmental conditions, standardized diet some days before the measurement) would be desirable in order to predict REE of master athletes more precisely than previously possible.

## Data Availability Statement

The original contributions presented in the study are included in the article/supplementary material, further inquiries can be directed to the corresponding author/s.

## Ethics Statement

The studies involving human participants were reviewed and approved by Ärtzekammer Nordrhein, Düsseldorf, Germany. The patients/participants provided their written informed consent to participate in this study.

## Author Contributions

PF-M, NR, WS, SM, EM, and FH carried out the experiment. PF-M and SH wrote the manuscript with support from MB and JR. PF-M and SH performed the analytical calculations. HT, PC, JA, and JR helped supervise the project. UM and WS managed the data. PF-M and MB conceived the original idea. All authors discussed the results and contributed to the final manuscript.

## Conflict of Interest

The authors declare that the research was conducted in the absence of any commercial or financial relationships that could be construed as a potential conflict of interest.

## Footnotes

1 www.drks.de
